# Ambient Air Pollution and Hospital Admissions for Peptic Ulcers in Taipei: A Time-Stratified Case-Crossover Study

**DOI:** 10.3390/ijerph16111916

**Published:** 2019-05-30

**Authors:** Shang-Shyue Tsai, Hui-Fen Chiu, Chun-Yuh Yang

**Affiliations:** 1 Department of Healthcare Administration, I-Shou University, Kaohsiung 82445, Taiwan; sionghak@isu.edu.tw; 2 Department of Pharmacology, College of Medicine, Kaohsiung Medical University, Kaohsiung 80708, Taiwan; chiu358@yahoo.com.tw; 3 Department of Public Health, College of Health Sciences, Kaohsiung Medical University, Kaohsiung 80708, Taiwan; 4 National Institute of Environmental Health Sciences, National Health Research Institute, Miaoli 35053, Taiwan

**Keywords:** ozone, nitrogen dioxide, air pollution, peptic ulcer, case-crossover, hospital admissions

## Abstract

Very few studies have been performed to determine whether there is a relationship between air pollution and increases in hospitalizations for peptic ulcer, and for those that have occurred, their results may not be completely relevant to Taiwan, where the mixture of ambient air pollutants differ. We performed a time-stratified case-crossover study to investigate the possible association between air pollutant levels and hospital admissions for peptic ulcer in Taipei, Taiwan. To do this, we collected air pollution data from Taiwan's Environmental Protection Agency and hospital admissions for peptic ulcer data for the years 2009–2013 from Taiwan's National Health Insurance's research database. We used conditional logistic regression to analyze the possible association between the two, taking temperature and relative humidity into account. Risk was expressed as odds ratios and significance was expressed with 95% confidence intervals. In our single pollutant model, peptic ulcer admissions were significantly associated with all pollutants (PM_10_, PM_2.5_, SO_2_, NO_2_, CO, and O_3_) on warm days (>23 °C). On cool days (<23 °C), peptic ulcer admissions were significantly associated with PM_10_, NO_2_, and O_3_. In our two-pollutant models, peptic ulcer admissions were significantly associated NO_2_ and O_3_ when combined with each of the other pollutants on warm days, and with PM_10_, NO_2_, and O_3_ on cool days. It was concluded that the likelihood of peptic ulcer hospitalizations in Taipei rose significantly with increases in air pollutants during the study period.

## 1. Introduction

The association between both short- and long-term exposure to air pollutants and daily mortality and hospitalizations attributed to diseases of the cardiovascular system and diseases of the lungs is well known [1,2,3,4,5]. Sustained long-term exposure to air pollution has been found to substantially lower life expectancy [6,7,8]. 

Several years ago, no correlation had been found between air pollution and hospitalization for diseases of the gastrointestinal track [9,10]. The relationship was once considered so unlikely that some studies investigating the effect of air pollution used intestinal diseases as a negative control [11,12]. However, there has been a recent increased interest in the effects of atmospheric pollutants on digestive diseases, including enteritis [13], appendicitis [14,15,16], inflammatory bowel diseases [17,18], non-specific abdominal pain [19], and peptic ulcer disease [20,21,22]. 

A peptic ulcer is a disorder of the gastroduodenal mucosa in which the epithelial cells rupture, causing pain and bleeding, and sometimes perforation [22]. Use of non-steroidal anti-inflammatory drugs (NSAIDs) and low-dose aspirin as well as infection by *Helicobacter pylori* have been clearly associated with increased risk of this disease [23,24]. However, a significant proportion of peptic ulcer cases cannot be explained by these well-known risk factors [25,26]. Interestingly, gastrointestinal bleeding has been found to occur more frequently in winter months, a finding that suggests its incidence could be affected by environmental factors [27]. 

Exposure to air pollutants have been shown to alter intestinal immunity [28], increase intestinal permeability [29,30], and alter gut microbial composition [28,31], all factors that could contribute to the development of peptic ulcers. Three recent epidemiologic studies have directly examined the specific relationship between air pollution and hospital admissions for peptic ulcer [20,21,22]. The results of these studies may not, however, be clearly applied to Taiwan, a region where air pollutant mixtures may differ. Therefore, by collecting official air pollutant data and national health insurance claim data, we performed a case-crossover study to investigate the possible association between short-term levels of various air pollutants and daily hospital admissions for peptic ulcers in Taipei, Taiwan.

## 2. Materials and Methods

### 2.1. Taipei City

Taipei City, with a population of 2.64 million, is the largest urban area in Taiwan. Its climate is subtropical with an average temperature of 23 °C. Although its extensive subway was created to reduce traffic congestion and air pollution, vehicle exhaust, especially from automobiles and motorcycles, remain the city's primary source of air pollution [32]. 

### 2.2. Hospital Admission Data

For this study, we collected daily counts of hospital admissions (including emergency room admissions and scheduled admissions) with a primary diagnosis of peptic ulcer (ICD-9 531-532) from Taiwan’s 2009 to 2013 National Health Insurance (NHI)’s Research Database (NHIRD), which includes insurance claims for almost all people in Taiwan. Taiwan’s National Health Insurance program, which was implemented on March 1, 1995, provides compulsory universal health insurance to all of Taiwan’s residents, covering about 98% of the population who receive care from 93% of the medical institutions and private facilities on the island. The NHI hospital dataset includes data elements such as birth day, gender, the date of admissions and discharge, and the International Classification of Diseases, 9th revision, Clinical Modification (ICD-9 CM) code for each admission. This dataset has been used as the basis for many past epidemiological studies and has been proved to be accurate and reliable [33]. This dataset, as well as the quality of its data, has been described previously [34]. This study was reviewed and approved by the Institutional Review Board at Kaohsiung Medical University Hospital (KMUH-IRB-exempt-20130018). 

### 2.3. Air Pollution and Meteorological Data

As described previously [16], we collected air pollution data obtained from six fixed air quality monitoring stations located in Taipei from Taiwan's Environmental Protection Administration. These monitoring stations measured hourly data of six air pollutants, including carbon monoxide (CO) (by nondispersive infrared photometry), sulfur dioxide (SO_2_) (by ultraviolet fluorescence), particulate matter (PM_10_ by beta-ray absorption and PM_2.5_ by tapered element oscillating microbalance method), nitrogen dioxide (NO_2_) (by ultraviolet fluorescence), and ozone (O_3_) (by ultraviolet photometry). The completeness of hourly data in these monitoring stations was above 97% for all pollutants during the study period (the mean was 97.7%). Hourly air pollution data from each of the monitors were collected for each day to calculate the mean daily levels. The daily exposure to each air pollutant was estimated by averaging hourly data across the stations, and from these values we calculated the daily average concentrations. We used the mean concentrations from the monitoring stations to represent the daily levels of Taipei City. Meteorological data including daily mean temperature and humidity were provided by the Central Weather Bureau’s Taipei observatory.

### 2.4. Statistical Analysis

This study used a case-crossover design [35,36,37], a good alternative to Poisson time-series regression models for evaluating the health effects of short-term increases in various pollutants, including those found in ambient air [35,38]. 

The study divided the observation period into several months. In this time-stratified approach [38], days falling on the same day of the week as the index day were defined as referent days in each month. We compared the case-period levels of various air pollutants with levels of those pollutants on all referent days. This approach is thought to reduce bias due to the non-stationarity of air pollution data [39,40]. In addition, the case-crossover design and the time-series approach yielded almost identical results [41,42]. Exposure to air pollutants was measured using the 2-day lagged cumulative moving average of air pollutant levels because previous studies have associated increased hospital admissions with high air pollutant levels on the same day of admission or over the previous two days [43,44]. Taipei City has a subtropical climate with no clear four-season cycle [32]. Hence, in this study, the possible interaction of seasonality on the effects of air pollutants was not considered; however, temperature was used instead. We analyzed our data by warm days (days with a mean temperature above 23 °C) and cool days (days with a mean temperature below 23 °C) only.

We performed conditional logistic regression to analyze the relationship between air pollutant levels and hospitalizations. Weights were equal to admissions counts on a given day. Risk was expressed as odds ratio (ORs) and significance was expressed as 95% confidence intervals (CIs). Exposure levels were entered as continuous variables. Meteorologic variables, such as temperature and humidity, which might play a confounding role, were considered in analyses. We adjusted for same-day average temperatures and humidity in the model. In all analyses, we modeled the same-day average temperature (lag 0) as a quadratic function, same-day average humidity (lag 0), and pollutants (the 3-day moving average from current day to previous 2 days concentration) as a linear function. ORs for the various air pollutants were calculated for increases in the interquartile range (IQR; between the 25th and the 75th percentiles). All statistical analyses were performed with the aid of SAS (version 9.4; SAS Institute, Inc., Cary, NC, USA).

## 3. Results

In total 23,205 patients were admitted for peptic ulcers in Taipei during the 5-year study period. As can be seen in Table 1, a summary of admissions characteristics and corresponding environmental data, peptic ulcer admissions averaged 12.71 per day over the entire study period, among which 62.54% were gastric ulcer and 37.46% were duodenal ulcer; 62.79% of the admissions were male and 37.21% were female. The average age was 65.77 ± 17.71 years. The mean daily count of admissions were 0.09, 5.37, 2.44, and 4.83 for ages <15, 15–64, 65–74, and >74 years, respectively. 

Pearson correlation coefficients among the air pollutants are presented in Table 2. A single pollutant model was used to analyze the associations between various ambient air pollutant levels and hospital admissions for peptic ulcer stratified by temperature (Table 3). Increases in the IQRs of all pollutants (PM_10_, PM_2.5_, SO_2_, NO_2_, CO, and O_3_) were significantly associated with increased risk of hospital admissions on warm days (>23 °C). However, on cool days (<23 °C), only increases in PM_10_, NO_2_, and O_3_ IQRs were found to be significantly associated with increased risk of hospital admission.

We used models including two pollutants to determine the independent effect of individual pollutants on hospitalizations for peptic ulcer (Table 4). This was performed to determine whether the effects of a particular pollutant would remain significant after each of the other pollutants was included in the model. On warm days, exposure to NO_2_ and O_3_ remained significantly associated with peptic ulcer admissions when combined with each of the other pollutants. PM_10_, NO_2_, and O_3_ remained significant in all two-pollutant models on cool days. The effect of SO_2_ remained significantly negative after incorporation of other pollutants into the model. 

## 4. Discussion

This study found significant positive relationships between ambient air NO_2_ and O_3_ levels and increased daily admissions for peptic ulcer on warm days and between PM_10_, NO_2_, and O_3_ and increased admissions on cool days. 

In this study, there was a significant negative effect of SO_2_ after the inclusion of other pollutants on cool days. We found no explanation for this in the literature, so it is possible that this inverse association was due to chance in the multiple significance testing process. It is also likely to be due to the collinearity between SO_2_ concentrations and other pollutant levels, which is a common problem in this type of study. 

Investigations on the influence of air pollution on admissions for peptic ulcers are rare. Quan et al. [20] conducted a case-crossover study in Calgary and Edmonton, Canada. They reported that short-term increases in the level of ambient air pollutants did not increase the incidence of upper gastrointestinal bleeding secondary to peptic ulcer disease. This study only identified 2523 cases in adult residents. The small sample size might have resulted in low statistical power to detect an association. The study of Wong et al. [21], which followed 66,820 elderly persons in order to determine the effects of long-term exposure to PM_2.5_ on hospital admissions for peptic ulcer in Hong Kong, found the hazard ratio to be 1.18 (95% CI = 1.02–1.36%) for peptic ulcer hospitalization per each 10 ug/m^3^ increase in PM_2.5_. Tian et al [22] conducted a study of this association among the elderly population in Hong Kong. They reported a 7.6% (95% CI = 2.2–13.2%) increase in emergency admissions for peptic ulcer bleeding for each IQR increment (25.8 ug/m^3^) in the 5-day moving average of NO_2_ concentration. Other pollutants (PM_2.5_, SO_2_, O_3_) were not associated with peptic ulcer bleeding admissions in that study. In our current study of Taipei City, hospital admissions for peptic ulcer rose with increases in NO_2_ and O_3_ concentrations on warm days. We also found an association between PM_10_, NO_2_, and O_3_ and increases in daily peptic ulcer admissions on cool days. Bleeding is a common complication of peptic ulcer diseases. It is estimated that 40% of hospital admissions for peptic ulcer were due to bleeding [22]. In this regard, we postulate that short-term elevation in air pollution might trigger peptic ulcer bleeding events and increase the risk of hospital admissions for peptic ulcer, although the percentage of all hospitalizations for peptic ulcer that were due to bleeding is unknown in this study. 

In this study, effects were observed on both warm and cool days, but they were larger on warm days (effect modification). The observed variation in effect estimates could be explained by variation in exposure patterns. People in Taipei are more likely to go outdoors and open the windows on warm days than on cool days (higher exposure); thus, monitored air pollutant levels may be closer to personal exposure on the warm days than on the cool days (better exposure assessment). This fact may reduce the air pollution effect on the cool days. On the other hand, differences in air pollution mixture between warm and cool days may also affect the effect estimates. Nevertheless, the degree to which variability and patterns of temperature may impact air pollution health effects needs to be further investigated. 

Air pollutants are well known to directly affect the respiratory and circulation systems, but we can only hypothesize about possible pathophysiological processes underlying the association between short-term exposure and the development of and admissions for peptic ulcers. Studies have suggested that air pollutants can enter the aerodigestive tract via mucociliary clearance of PM from the lungs and can also enter that tract via ingestion of contaminated food and water [45,46,47]. Those studies also have suggested that the inhaled air pollutants may have direct toxic effects on epithelial cells after they have been transported to that location [45,46,47]. Still another explanation may be related to the fact that air pollutants increase intestinal permeability, alter the gut microbiota, and promote inflammation, thus contributing towards the development of peptic ulcer diseases in these ways [28,29,30,31]. Colonic microflora and elevated interleukin (IL)-8 and IL-17 levels in the small and large intestines have been found to be altered in mice guts exposed to PM [28]. In humans, exposure to O_3_ can stimulate the production of tumor necrosis factor, IL-6, and IL-8 and induce systemic pro-inflammatory responses [48,49]. Such inflammatory responses, caused by disturbances in microbe composition in the gut and metabolic processing, have been found in mice fed PM [31]. Thus, exposure to air pollution, regardless of whether it is inhaled or ingested orally, may cause an imbalance in gut microbiota, thereby inducing an inflammatory response, which can lead to peptic ulcer. In addition, gaseous pollutants have been related to systemic inflammation, which may further induce or worsen adverse effects in the gastrointestinal tracts [50].

A case-crossover study design is a good approach to determining the health risk prompted by acute and intermittent exposures to possibly toxic substances. This design makes it possible to control for many confounders without the need for additional statistical modelling. All comparisons are within-individuals, so the design reduces confounding by time-invariant individual variables, including gender, age, underlying chronic diseases, and *Helicobacter pylori* infection. In addition, the time stratification approach we used in this study has been found to effectively control for seasonality, time trends, and chronic diseases and confounders that vary slowly over time [39,40]. 

We determined levels of exposure to air pollutants based on averaged data collected by fixed monitoring stations, which do not capture personal levels of exposure. The levels across various stations were averaged to access the population exposure levels to air pollution assuming that exposure was homogeneous throughout the area studied. This may raise some concerns over our estimates, especially since concentrations may differ between monitoring stations [51]. There may have been some errors in the measurement of exposure due to differences between population-averaged exposure and ambient air pollutant levels. This lack of individual measurements may have led to some misclassification and non-differential bias. However, this would produce an effect close to the null, resulting in an underestimation of the association [43,52].

There are some limitations with our study design. One limitation is that it is limited to one subtropical city. Another is that the peptic ulcer patients in this study include both the acute and the chronic types as well as both those with and without bleeding—patients presenting with different types of peptic ulcers and/or complications associated with peptic ulcers may have differences in symptom onset and/or delays of presentation to hospital [20,22]. This misclassification, however, was likely to be non-differential, which would tend to underestimate rather than overestimate the association. Another limitation is that our population belonged to the same race and ethnic background. Both of these limitations may reduce the possibility of generalizing our findings to other study areas. Another is that we did not take into account lifestyle behaviors, including how the use of air conditioning or time spent outdoors may affect personal exposures, which may vary widely with those of other study populations and locations. The design used in this study could have some problems with confounding by time-dependent covariates, such as the use of NSAIDs or aspirin. However, since the control days were the same day of the week in the same month, the differences in NSAIDs usage would likely not differ much over a short time period within the month studied [22]. Additionally, it is unlikely that day-to-day variation in NSAIDs usage would be correlated with the different levels of air pollutants. 

## 5. Conclusions

In summary, in Taipei, Taiwan, short-term exposure to certain air pollutants (PM_10_, NO_2_, and O_3_) is significantly associated with increased risk of hospital admissions due to peptic ulcers.

## Figures and Tables

**Table 1:** Distribution of daily peptic ulcer admissions, weather, and air pollutant concentrations in Taipei, Taiwan, 2009–2013.

	Median ^a^	Mean	Maximum
PM_10_ (ug/m^3^)	42.51 (31.21–57.28)	47.09	205.35
PM_2.5_ (ug/m^3^)	25.24 (18.59–34.69)	27.80	140.54
SO_2_ (ppb)	2.80 (2.11–3.82)	3.08	9.04
NO_2_ (ppb)	22.01 (18.22–26.40)	22.73	61.94
CO (ppm)	0.56 (0.44–0.71)	0.61	1.99
O_3_ (ppb)	23.29 (17.74–30.57)	24.69	63.15
Temperature (°C)	23.91 (19.08–28.60)	23.48	33.18
Humidity (%)	73.17 (66.64–80.35)	73.06	93.78
Daily hospital admissions Peptic ulcer (*n* = 23,205)	12 (10–16)	12.71	32
Gastric ulcer (*n* = 14,513)	8 (5–10)	7.96	20
Duodenal ulcer (*n* = 8692)	5 (3–6)	4.77	15
Male (*n* = 14,571)	8 (6–10)	7.99	21
Female (*n* = 8634)	4 (3–6)	4.73	17
Age <15 (*n* = 163)	0 (0–0)	0.09	3
Age 15–64 (*n* = 9787)	5 (3–7)	5.37	17
Age 65–74 (*n* = 4453)	2 (1–3)	2.44	11
Age >74 (*n* = 8802)	5 (3–6)	4.83	15

^a^ median (25–75 percentile).

**Table 2:** Pearson correlation coefficients among air pollutants.

Variable	PM_2.5_	PM_10_	NO_2_	CO	O_3_	SO_2_
PM_2.5_	1.0	0.78	0.57	0.57	0.28	0.64
PM_10_		1.0	0.33	0.36	0.25	0.42
NO_2_			1.0	0.90	−0.01	0.56
CO				1.0	−0.27	0.55
O_3_					1.0	−0.01
SO_2_						1.0

**Table 3:** Association between exposure to air pollutant and hospital admissions for peptic ulcer in a single pollutant model for Taipei, Taiwan, 2009–2013.

Pollutant	Whole period (*n* = 23,205)	>23 °C (1001 days) (*n* = 12,101)	<23 °C (825 days) (*n* = 11,104)
	OR (95% CI) ^a,b^	OR (95% CI) ^a,b^	OR (95% CI) ^a,b^
PM_10_	1.00 (0.98–1.02)	1.05 (1.01–1.08) ^c^	1.04 (1.02–1.07) ^c^
PM_2.5_	1.00 (0.98–1.03)	1.14 (1.09–1.18) ^c^	1.01 (0.98–1.04)
SO_2_	1.00 (0.98–1.02)	1.04 (1.00–1.08) ^c^	0.92 (0.89–0.95)
NO_2_	1.00 (0.98–1.02)	1.16 (1.12–1.20) ^c^	1.07 (1.04–1.11) ^c^
CO	1.00 (0.98–1.02)	1.17 (1.12–1.21) ^c^	1.02 (0.99–1.05)
O_3_	1.00 (0.97–1.03)	1.11 (1.07–1.15) ^c^	1.23 (1.17–1.28) ^c^

^a^ Interquartile range (IQR): PM_10_ (26.07 μg/m^3^), PM_2.5_ (16.1 μg/m^3^), SO_2_ (1.71 ppb), NO_2_ (8.18 ppb), CO (0.27 ppm), and O_3_ (12.83 ppb); ^b^ control for temperature and humidity; ^c^ *p* < 0.05.

**Table 4:** Risk of peptic ulcer admissions per IQR increase in two-pollutant model by temperature ^a,b^ .

	Control for PM_10_	Control for PM_2.5_	Control for SO_2_	Control for NO_2_	Control for CO	Control for O_3_
	OR (95% CI)	OR (95% CI)	OR (95% CI)	OR (95% CI)	OR (95% CI)	OR (95% CI)
PM_10_						
>23 °C	–	–	1.04 (1.00–1.08)	0.98 (0.94–1.02)	0.98 (0.94–1.02)	1.02 (0.98–1.05)
<23 °C	–	–	1.09 (1.06–1.11)	1.03 (1.01–1.06)	1.05 (1.02–1.07)	1.05 (1.03–1.07)
PM_2.5_						
>23 °C	–	–	1.18 (1.12–1.24)	1.03 (0.97–1.09)	1.04 (0.99–1.10)	1.10 (1.05–1.15)
<23 °C	–	–	1.13 (1.08–1.18)	0.96 (0.93–1.00)	0.99 (0.96–1.04)	1.02 (0.99–1.06)
SO_2_						
>23 °C	1.02 (0.98–1.06)	0.95 (0.91–0.99)	–	0.90 (0.86–0.95)	0.96 (0.92–0.99)	1.01 (0.97–1.05)
<23 °C	0.87 (0.84–0.91)	0.84 (0.81–0.88)	–	0.81 (0.77–0.84)	0.84 (0.80–0.88)	0.97 (0.93–1.00)
NO_2_						
>23 °C	1.17 (1.12–1.22)	1.14 (1.07–1.19)	1.24 (1.18–1.30)	–	1.10 (1.02–1.18)	1.14 (1.10–1.18)
<23 °C	1.06 (1.02–1.09)	1.09 (1.05–1.13)	1.22 (1.17–1.27)	–	1.28 (1.20–1.37)	1.25 (1.20–1.30)
CO						
>23 °C	1.18 (1.13–1.23)	1.14 (1.09–1.20)	1.20 (1.14–1.25)	1.07 (0.99–1.16)	–	1.16 (1.12–1.21)
<23 °C	0.99 (0.96–1.03)	1.02 (0.98–1.06)	1.14 (1.09–1.19)	0.82 (0.77–0.88)	–	1.17 (1.12–1.21)
O_3_						
>23 °C	1.10 (1.06–1.15)	1.06 (1.01–1.11)	1.11 (1.06–1.16)	1.07 (1.03–1.12)	1.10 (1.06–1.15)	–
<23 °C	1.23 (1.18–1.29)	1.23 (1.18–1.29)	1.21 (1.15–1.26)	1.47 (1.39–1.55)	1.40 (1.32–1.48)	–

^a^ IQR: PM_10_ (26.07 μg/m^3^), PM_2.5_ (16.1 μg/m^3^), SO_2_ (1.71 ppb), NO_2_ (8.18 ppb), CO (0.27 ppm), and O_3_ (12.83 ppb); ^b^ Control for temperature and humidity.
